# Complex emergencies and the control and elimination of neglected tropical diseases in Africa: developing a practical approach for implementing safe and effective mapping and intervention strategies

**DOI:** 10.1186/s13031-021-00356-7

**Published:** 2021-03-31

**Authors:** Louise A. Kelly-Hope, Angelia M. Sanders, Emma Harding-Esch, Johan Willems, Fatima Ahmed, Fiona Vincer, Rebecca Hill

**Affiliations:** 1 Liverpool School of Tropical Medicine, Liverpool, UK; 2 The Carter Center, Atlanta, USA; 3 London School of Hygiene & Tropical Medicine, London, UK; 4 CBM, Bensheim, Germany; 5 The MENTOR Initiative, Haywards Heath, UK; 6 Sightsavers, Haywards Heath, UK

**Keywords:** Neglected tropical diseases, NTDs, Sub-Saharan Africa, Conflict, Crisis, Complex emergencies, Mapping, Mass drug administration, MDA, South Sudan, Sustainable development goals, SDGs, Lymphatic filariasis, Onchocerciasis, Schistosomiasis, Soil transmitted helminths, Trachoma

## Abstract

**Background:**

Complex emergencies resulting from conflict and political instability are a major challenge for national neglected tropical diseases (NTDs) control and elimination programmes, especially in sub-Saharan Africa. Currently, there are no formal guidelines for national programmes to use and plan activities in these humanitarian situations, therefore the aim of this study was to develop a new methodological approach for making decisions about the implementation of safe and effective mapping and mass drug administration (MDA) intervention strategies.

**Methods:**

The study focussed on the 47 World Health Organization’s African Region (AFR) countries. NTD data were based on five diseases controlled by preventive chemotherapy (PC; i.e. lymphatic filariasis, onchocerciasis, schistosomiasis, soil transmitted helminths, trachoma), obtained from the WHO data portals and The Global Trachoma Atlas for 2018. Data on complex emergencies were obtained from the Armed Conflict Location and Event Data Project for 2018–2019.

NTD and conflict data were summarised and mapped. A decision tree was developed using NTD mapping, endemicity, MDA and implementing partners data, together with conflict status information at district level. South Sudan was used as a case study, given its current nexus of high NTD prevalence, incidences of conflict, and the presence of a national NTD programme and supporting partners.

**Results:**

For the five NTDs, between 26 and 41 countries required PC, 69.2–212.7 million people were treated with coverage between 54.8–71.4%. In total 15,273 conflict events were reported including high rates of violence against civilians (29.4%), protests (28.8%), and battles (18.1%). The decision tree process included four main steps including i) information gathering ii) determine a disease mapping strategy iii) determine an MDA implementation strategy and iv) create a disease and conflict database. Based on these steps, risk maps were created. The South Sudan case study on onchocerciasis found the majority of the districts requiring mapping or MDA had a conflict event, and required specialised methods adapted to context and risk, with support from implementation partners in selected areas.

**Conclusions:**

The paper presents a new methodological approach for implementing safe and effective mapping and intervention strategies in NTD endemic countries with ongoing complex emergencies, which will help to address challenges and make progress toward the NTD Roadmap targets of 2030.

**Supplementary Information:**

The online version contains supplementary material available at 10.1186/s13031-021-00356-7.

## Background

Complex emergencies are humanitarian crises resulting from conflict and political instability [1–4]. They are often tied to social inequities, inequalities, and poverty and can lead to disruption of livelihoods, threats to life and large-scale movement and displacement of people. These complex emergencies have a negative impact on the health of affected populations, with most deaths occurring due to preventable causes such as infectious diseases and malnutrition. Further, they can impede large-scale disease control and elimination programmes such as those related to neglected tropical diseases (NTDs) [5–9]. NTDs are a group of poverty-related diseases, that are often stigmatising, impact individuals’ social and economic contributions [10], and are currently a global health priority for elimination [11, 12]. Several of these diseases can be controlled or eliminated through interventions including preventive chemotherapy (often distributed as mass drug administration (MDA)) and efforts to improve sanitation and hygiene.

The global NTD community marked out an ambitious goal of eliminating NTDs through the 2012 London Declaration on NTDs [13]. This was inspired by the World Health Organization’s (WHO) 2020 Roadmap on NTDs [14], and though progress has been made in multiple countries in reducing their burden, the global goals of the future Roadmap 2021–2030 [11] will be hindered if the challenge of tackling NTDs in complex emergencies is not addressed. Those living in complex emergencies are at an increased risk of NTDs due to factors increasing susceptibility and exposure to infection and disease as health infrastructure, human resources and programmes are destabilised, and funding is redirected [7–9, 15–17]. Currently there are no WHO guidelines on how endemic country programmes may address these significant challenges, which has the potential to ‘leave them behind’.

Understanding the type, magnitude and location of conflicts that affect NTD activities is crucial in helping national NTD programmes and their supporting partners adapt their programmes to account for populations that would not typically be within their scope of work, such as refugees and internally displaced people (IDPs). Several approaches and examples to address these problems have been suggested, including adaptive programming, flexible funding, opportunistic partnerships and learning from organisations with experience in conflict zones [8].

Currently there is limited documentation on the impact of complex emergencies in the context of preventive chemotherapy (PC) for NTDs and the ability of national programmes to map endemicity and implement interventions such as MDA [17]. This is of particular concern in sub-Saharan African, where numerous countries have among the highest prevalence of NTDs combined with the lowest levels of peace, including, for example, South Sudan, Central African Republic and Democratic Republic of Congo [9, 18, 19]. This may be further exacerbated with the emergence and spread of Covid-19 in conflict zones, adding further complexity to the Sustainable Development Goals (SDGs) pledge to ‘leave no one behind’ [20–22].

To effectively implement an NTD programme, it is important to know where the different diseases are endemic through baseline mapping, how prevalent these diseases are, whether interventions are in place and how effective they have been, and if there are implementing partners, often non-governmental organisations (NGOs), already operating effectively in those locations. The WHO NTD PC databank and Expanded Special Project for Elimination of NTDs (ESPEN) data portal [23] allow health ministries and stakeholders to share programme data, and provide important starting points for this information in African NTD-endemic countries. This information can be used together with data on complex emergencies available from data portals such as the Armed Conflict Location and Event Data Project (ACLED) [24, 25], to help make informative programmatic decisions.

The NTD NGO Network (NNN) has a Conflict and Humanitarian Emergency (C&HE) Cross-Cutting Group comprising a diverse range of NTD experts [26]. It acknowledges that the implementation of NTD mapping and interventions in complex emergencies is difficult and made more challenging given that there are no current guidelines to support endemic countries. Therefore, the NNN C&HE Group - Mapping Task Team aimed to develop methodological approach for making decisions about the implementation of safe and effective mapping and MDA intervention strategies in the WHO African Region (AFR). To demonstrate an example of this new approach, a case study in South Sudan is presented given its current nexus of high NTD prevalence, the protracted and ongoing nature of conflict, low levels of peace and the presence of a national NTD programme and supporting partners.

## Methods

Data on NTDs and conflict events in the 47 AFR countries, including 17 countries in West Africa, 10 in Central Africa, 10 in Eastern Africa and 10 in Southern Africa were considered in unison to better understand the context and develop a stepwise decision tree to facilitate new mapping and implementation strategies. The method section is broken down to show how the data sources and summaries can be consolidated, with selected aspects included in the decision tree and risk map. A case study country is presented as an example.

### Data sources and summaries

#### NTDs

The PC NTDs controlled and eliminated through MDA were used as examples in this new methodology, and included lymphatic filariasis (LF), onchocerciasis, schistosomiasis, soil-transmitted helminths (STH), and trachoma. First, the status of PC for the five NTDs in AFR for the latest year available (2018) from the latest WHO update on the global status of PC implementation [27] were summarised by the number of people requiring PC, number of countries that implemented and reported activities, proportion of districts that implemented PC, and proportion of districts achieving effective coverage. Second, national level coverage data for the latest year available (2018) were obtained from the WHO Global Health Observatory / PC data portal [28] and mean PC coverage summarised for each AFR sub-region [10]. Finally, the WHO ESPEN data portal [23] was used to highlight the AFR, national and subnational data available on mapping, endemicity, MDA and implementing partner status for LF, onchocerciasis, schistosomiasis, STH, and used for the South Sudan case study. The Global Trachoma Atlas was used to highlight the endemicity of trachoma in Africa [29].

#### Complex emergencies

Information was based on conflict data obtained from ACLED [24], which is a freely available tracking project that plots geo-referenced location data, including conflict event types, specifically battles, riots, protests, strategic developments, explosions/remote violence and violence against civilians (conflict type definitions are summarised in Additional Table 1). ACLED data were used as they were available for the majority of NTD endemic countries and summarised by the AFR sub-regions for the 12 months from 1st September 2018 to 31st August 2019. Maps showing the location of event types were created in ArcGIS 10.7 (ESRI, Redlands, CA). Administrative boundaries were obtained from the Humanitarian Data Exchange (HDX) [30]. The ACLED data were primarily used as the geo-referenced standardised data allowed better comparisons across countries.

### Decision tree and risk map development

A decision tree was developed taking into account the status of NTD i) mapping ii) endemicity iii) MDA and iv) implementing partners, together with v) conflict data at district level or the administrative implementing area, which may also include a county or health zone, and be referred to as an implementation unit (IU) or evaluation unit (EU) depending on the NTD and/or activity being conducted. For example, LF mapping and MDA activities are measured using IUs and post-MDA surveillance using EUs, whereas trachoma mapping and MDA activities are measured using EUs. For the purpose of this paper we are using ‘district’ to denote the implementing unit (i.e. district, county, health zone, IU, EU).

Four key steps were defined to facilitate the collation and classification of the NTD and conflict data, the creation of a new combined database, and the development of the decision tree and related risk maps for each district/implementing area. These include i) information gathering ii) determine a disease mapping strategy iii) determine an MDA implementation strategy and iv) create a disease and conflict database.

### Case study

Using this methodological approach, South Sudan is presented as a case study as it has one of the highest burdens of NTDs [10] and lowest levels of peace with a ranking of 161 out of 163 on the global peace index [19]. Due to widespread conflict since 2013, more than four million people have been displaced from their homes rendering them as either refugees or IDPs. Despite these challenges, South Sudan has an active NTD programme with ongoing activities in parts of the country where disease prevalence is known [23].

In order for the NTD programme to expand its mapping and implementation activities, it needs to consider them in the context of the ongoing conflict events across the country. In total there are 10 States with 79 districts used as IUs in South Sudan [23].

The new decision tree outlined below was trialled to examine one NTD (onchocerciasis) as a case study in order to help inform and expand safe, effective disease mapping and MDA implementation. First, the NTD and ACLED conflict data for South Sudan were summarised to provide an overview of the current situation. Second, onchocerciasis mapping, endemicity, MDA and implementing partner data were collated and classified for each district. Third, the mapping and MDA implementation strategy were determined to create related mapping and MDA maps.

Finally, the decision tree and South Sudan case study were presented at two interactive sessions of the annual NNN Conference in September 2019 by members of the C&HE Group – Mapping Task Team to assess practicality and acceptability among participants who work in NTD endemic countries, which may be vulnerable to complex emergencies [26].

## Results

### NTD summaries

The WHO update on the global status of implementation of PC for the five PC NTDs in AFR in 2018 reported that the number of countries requiring PC ranged between 26 and 41; number of people requiring PC ranged between 110 and 342 million; number of countries that implemented and reported activities between 18 and 30, percentage of districts that implemented PC between 7.3–86.4% (excluding pre-school aged children (PAC) for STH and trachoma); percentage of districts achieving effective coverage between 47.1–90.3% (excluding PAC for STH and trachoma); number of people in need and treated (millions) between 69.2–212.7 and overall coverage between 54.8–71.4% (Table 1).

**Table 1:** Status of preventive chemotherapy for five NTDs in the WHO African Region in 2018

PC implementation	LF	Oncho	Schistosomiasis SAC^**6**^	STH SAC^**6**^	Trachoma
Number of countries requiring PC^1^	32	26	41	40	26
Number of people requiring PC (millions)	342.3	215.3	109.8	176.5	157.8
Number of countries implemented and reported activities^2^	25	23	29	30	18
Proportion (%) of districts implemented PC^3^	78.3	86.4	39.8	67.8	NR^7^
Proportion (%) of districts achieving effective coverage^4^	89.8	90.3	88.9	85.8	NR^7^
Number of people in need and treated (millions)	212.7	153.7	69.2	114	86.4
Coverage (%)^5^	62.1	71.4	63.1	64.6	54.8

Table adapted from information in the WHO update on the global status of implementation of preventive chemotherapy (PC) [27].

^1^Number of endemic countries moved to post-treatment surveillance stage is not included in total.

^2^Number of countries reporting data on PC implementation. Countries submitting blank reports are not included in total.

^3^Proportion of known endemic districts implementing PC for SAC in countries that reported on PC interventions.

^4^Proportion of districts implementing PC achieving the defined effective coverage of SAC population for the disease - > 65% for LF and Onchocerciasis; > 75% for schistosomiasis and STH; > 80% for trachoma.

^5^Coverage is calculated as the number of people in need of PC and treated out of total population requiring PC.

^6^SAC – school-aged children.

^7^NR - not reported.

The WHO Global Health Observatory PC data for AFR countries requiring PC in 2018 were summarised for each country (available in Additional Table 2). The mean coverage rates for each of the five PC NTDs in the four AFR regions in 2018 are summarised in Table 2 and highlights that mean rates for LF ranged from 39% (Central) to 65.8% (Western), for onchocerciasis from 48.1% (Central) to 82.8% (Southern), for schistosomiasis SAC 22.1/8.1% (Southern) to 71/27.5% (Western), for STH SAC from 5.7/42.3% (Southern) to 42.1/62.4% (Western) and for trachoma 15.5% (Western) to 26.7% (Eastern).

**Table 2:** Mean coverage of preventive chemotherapy for five NTDs in the WHO African sub-regions in 2018

PC implementation	LF ***n*** = 32	Oncho ***n*** = 25	Schistosomiasis SAC^**1**^ ***n*** = 42	STH SAC^**1**^ ***n*** = 40	Trachoma ***n*** = 27
Southern Africa	58.1	82.8	22.1	42.3	16.3
Eastern Africa	43.0	71.1	50.3	50.1	26.7
Central Africa	39.0	48.1	59.8	48.1	18.7
Western Africa	65.8	80.0	71.0	62.4	15.5

Note:^1^SAC – school-aged children.

Data source: Preventive chemotherapy (PC) Data Portal; Country profiles (28)

**Fig. 1:**
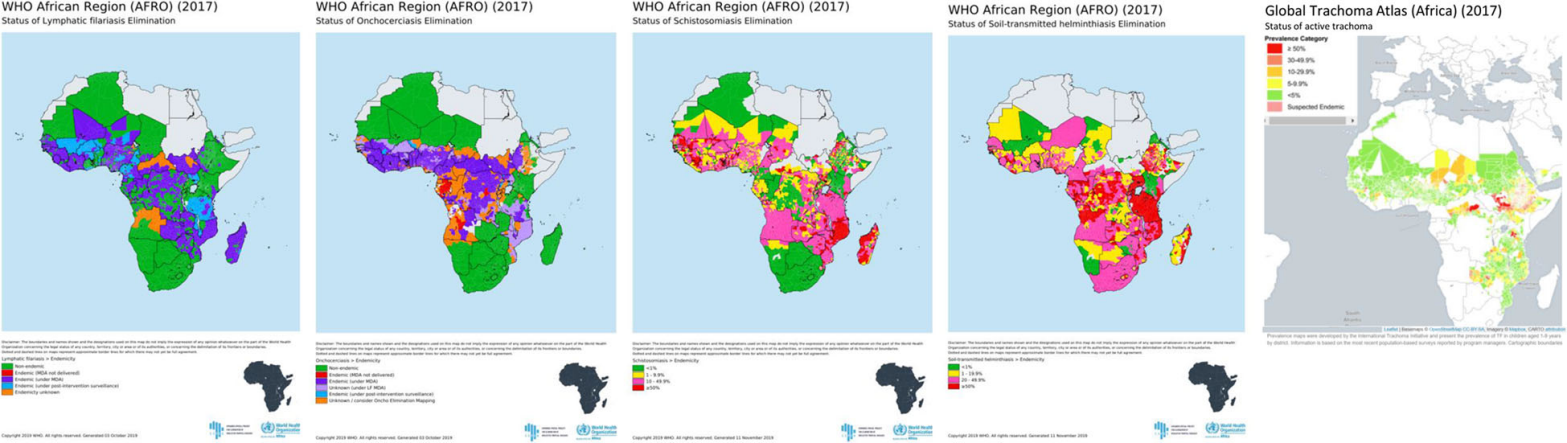
Maps of the WHO African Region disease elimination status for selected NTD in 2017. Map source: ESPEN [23]

The WHO ESPEN data portal district maps were used to highlight the status of elimination for LF, onchocerciasis, schistosomiasis, and STH, with the latest data available for 2017 as shown in Fig. 1 [23]. For LF and onchocerciasis, the status of endemicity is presented in relation to both endemicity and MDA status, whereas for schistosomiasis and STH, the status is presented in relation to prevalence. The Global Trachoma Atlas maps were used to highlight the prevalence of active trachoma for 2017.

### Conflict summaries

**Fig. 2:**
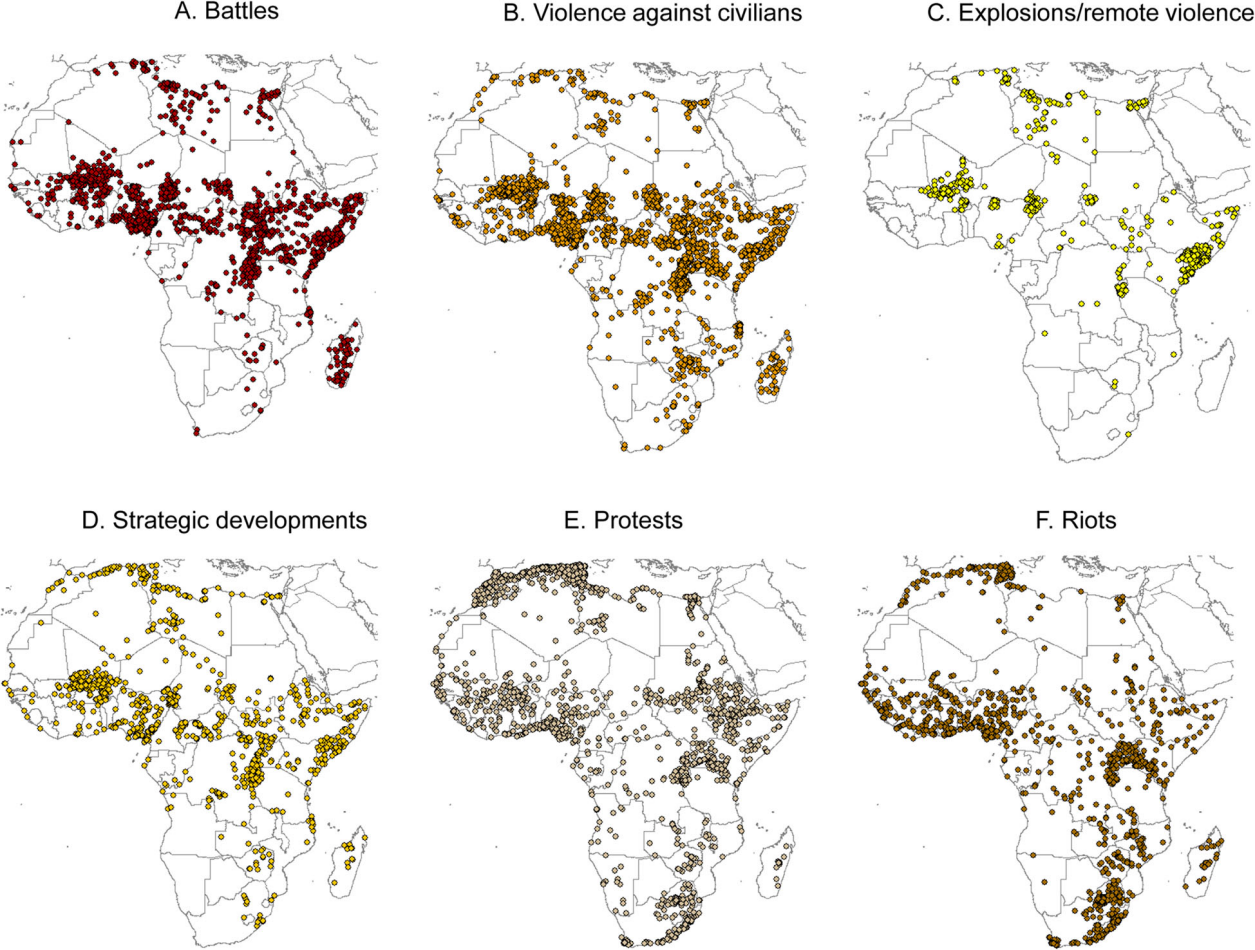
Location of reported conflict event types on the WHO African region, September 2018 – August 2019. Data source: ACLED [24]. Note: Maps include continental Africa; however, analysis focused on the AFR region which does not include North Africa

There was a total of 15,273 conflict events in the WHO AFR countries between September 2018 and August 2019, and the distribution of event types is shown in Fig. 2. The highest proportion of conflict events occurred in the Western region (43.7%), and the lowest proportion in the Southern region (12.5%). The most common event type was violence against civilians (29.4%), followed by protests (28.8%) and the least common was explosion/remote violence (2.5%) (Table 3).

**Table 3:** Summary of conflict event types by WHO AFR sub-region September 2018 – August 2019

Region	Battles	Explosion Remote violence	Protests	Riots	Strategic developments	Violence against civilians	Total by region (% of total)
Southern Africa	30	2	827	759	43	250	1911 (12.5)
Eastern Africa	517	28	359	271	152	770	2097 (13.7)
Central Africa	1199	84	561	309	540	1895	4588 (30.0)
Western Africa	1023	273	2647	648	518	1568	6677 (43.7)
Total events (% of total)	2769 (18.1)	387 (2.5)	4394 (28.8)	1987 (13.0)	1253 (8.2)	4483 (29.4)	15,273

Note: Data not available for São Tomé and Príncipe, Comoros, Seychelles and Cape Verde [24]

**Fig. 3:**
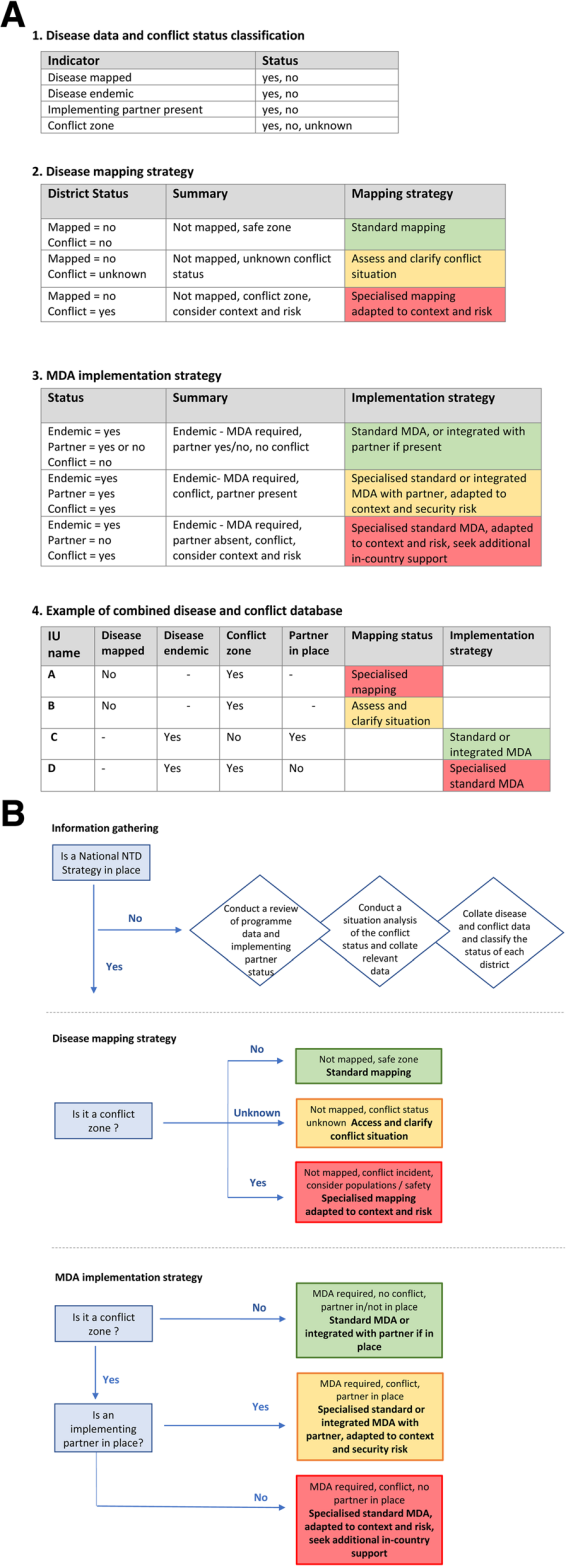
Disease and conflict data for mapping and/or implementation decision tree development

**TEXT BOX: Decision tree and risk map steps.**

**Table:** 

The four step -process for decision tree and related risk map development, outlined below and summarised in Fig. 3, was developed and applied to the South Sudan case study. Step 1. Information gathering: First, determine if a national NTD strategy is in place with information on the mapping and endemicity status of each district. If a strategy is not in place, then conduct a desk-based review of programme data and implementing partner status. Next, conduct a situation analysis of the conflict status and gather relevant information. Finally, collate all data on disease mapping, endemicity, implementing partners and conflict status into an Excel database and classify each district according to their status: disease mapped = yes, no; disease endemic = yes, no, unknown; implementing partner present = yes, no; Conflict zone = yes, no, unknown (Fig. 3a.1). Step 2. Determine a disease mapping strategy: For the districts that require mapping, the conflict status (yes, no, unknown) will determine the level of risk and strategy: i) ‘not mapped/no conflict’ indicates a safe zone and standard mapping can be conducted; ii) ‘not mapped/conflict unknown’ indicates further assessment required to clarify conflict situation before proceeding; iii) ‘not mapped/conflict yes’ indicates an unsafe zone and that specialised mapping adapted to the context and risk needs to be conducted (Fig. 3a.2). Step 3. Determine an MDA implementation strategy: For the districts that are endemic and require MDA, the combination of the implementing partner (yes, no) and conflict status (yes, no) will determine the level of risk and strategy including: i) ‘endemic/partner yes or no/conflict no’ indicates a safe zone, and standard or integrated (if partner present) MDA ii) ‘endemic/partner yes/conflict yes’ indicates unsafe zone, and that specialised standard or integrated MDA with partner, adapted to context and security risk, needs to be conducted; iii) ‘endemic/partner no/conflict yes/ indicates unsafe zone and that specialised standard MDA, adapted to context and security risk with additional in-country support, needs to be conducted (Fig. 3a.3). All three MDA strategies require collaboration with the NTD programme and/or implementing partners. 4. Create a disease and conflict database: For the districts that require mapping and/or MDA, the data needs to be combined into one database using the classifications of the mapping, endemicity, implementing partner and conflict status. The mapping and/or MDA implementation strategy needs to be recorded, to provide disease endemicity, MDA and conflict status overview, and help develop a country-specific decision tree and risk maps (Fig. 3a.4).	

### South Sudan case study for onchocerciasis

#### Information gathering of NTD programme data

LF, onchocerciasis, schistosomiasis, STH and trachoma were reported in South Sudan, which has a population of 9,991,337 (2016), with PC required for each of these NTDs. The mean MDA coverage rate for each disease in 2018 was low with LF reporting 17.4%, onchocerciasis 25.4%, schistosomiasis 0%, STH (SAC only) 40.7% and trachoma 13.5%. The WHO ESPEN portal provided data on endemicity programmatic requirements in 2017 and were summarised as the number of districts in each state requiring mapping or MDA implementation (Table 4). Of the 79 districts, the endemicity was known for LF in 78 districts (98.7%), onchocerciasis in 48 districts (60.8%), schistosomiasis in 39 districts (49.4%) and STH in 10 districts (12.7%), indicating extensive mapping still required for the latter three diseases. The Global Trachoma Atlas showed that trachoma endemicity was known in 29 districts (36%). Two implementing partners were reported in the ESPEN data portal including The MENTOR Initiative (10 districts for onchocerciasis) and The Carter Centre (5 districts for trachoma), and a further two reported by co-author (JW), including the WHO (4 districts for onchocerciasis) and CBM (4 districts for onchocerciasis). Overall, these implementing partners were supporting the NTD programme in 21 districts (details available in the Additional Table 3).

**Table 4:** Summary of the number of districts requiring mapping or MDA implementation by each state in South Sudan in 2017

State (number of districts)	LF		Oncho		Schisto*		STH*		Trachoma*	
	Map	MDA	Map	MDA	Map	MDA	Map	MDA	Map	MDA
Central Equatoria (*n* = 6)		6		6		6	1	5	4	2
Eastern Equatoria (*n* = 8)		8	4	4		8	3	5	3	5
Jonglei (*n* = 11)	1	10	8	3	8	3	11		5	6
Lakes (n = 8)		8		8		8	8		8	
Northern Bahr el Ghazal (*n* = 5)		5	2	3		2			5	
Unity (*n* = 9)		9	9			9	8			9
Upper Nile (*n* = 12)		12	8	4	12		12		8	4
Warrap (*n* = 7)		7		7	2	1	7		7	
Western Bahr el Ghazal (n = 3)		3		3	2	1	3		3	
Western Equatoria (*n* = 10)		10		10	8	1	10		7	
**Total**	**1**	**78**	**31**	**48**	**32**	**39**	**63**	**10**	50	**24**

Note: * Schistosomiasis - 8 districts non-endemic, STH – 5 districts non-endemic and Trachoma – 3 districts non-endemic

#### Situation of conflict status

**Fig. 4:**
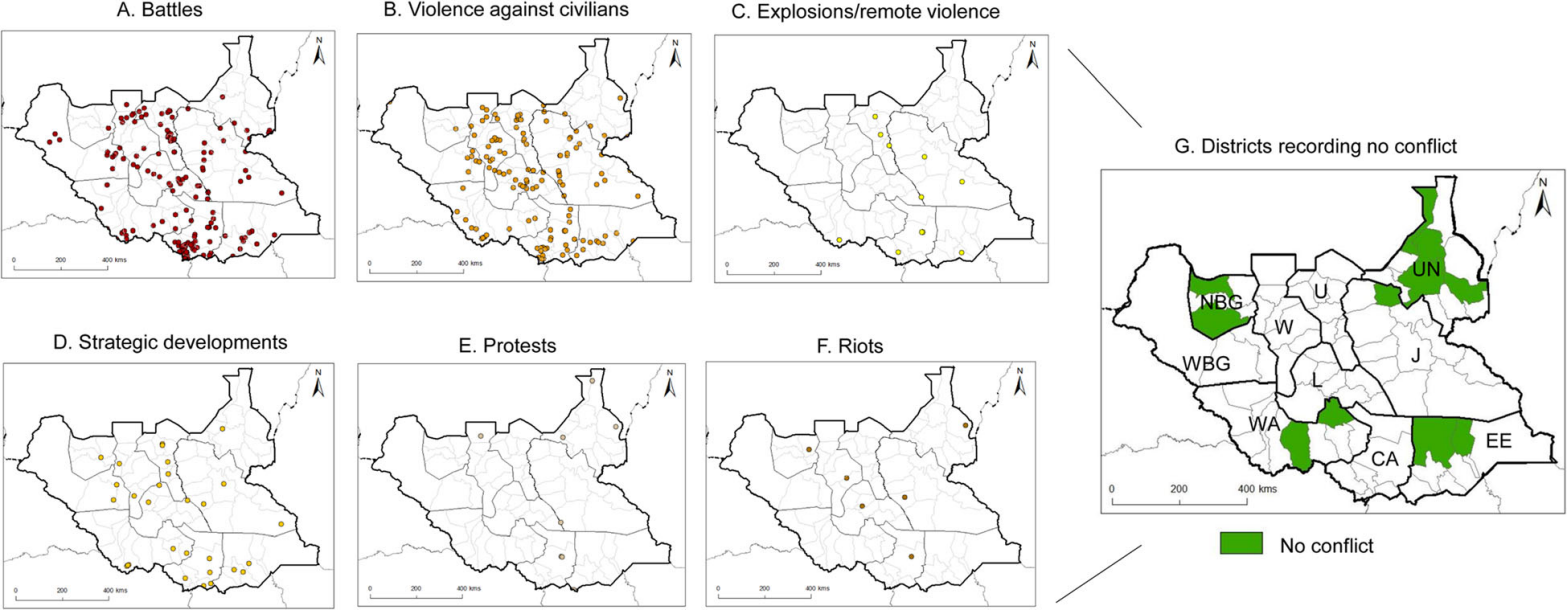
Location of recorded conflict events and districts affected in South Sudan September 2018 to August 2019. Data sources: ACLED [24] and Administrative boundary source: Humanitarian Data Exchange [30]. Note: State abbreviations include WBG-Western Bahrel Ghazal; NBG- Northern Bahrel Ghazal; W- Warrup; U – Unity; UN- Upper Nile; J-Jonglei; EE- Eastern Equatoria; CA – Central Equatoria; Western Equatoria

There was a total of 673 conflict events reported in South Sudan between September 2018 to August 2019 and the distribution of event types is shown in Fig. 4a-f. The highest proportion of conflict events occurred in the Central Equatorial region (39.4%), and the lowest proportion in the Northern Bahr el Ghazal region (0.7%). In total 68 (86.1%) of the 79 districts recorded at least one conflict event and 11 districts (13.9%) recorded no conflict event, as shown in Fig. 4g. The most common conflict type was violence against civilians (51.0%), followed by battles (36.6%) and the least common was explosion/remote violence (1.5%) (Table 5).

**Table 5:** Summary of the number of conflict event types in each states of South Sudan in 2018–2019

State	Battles	Explosion Remote violence	Protests	Riots	Strategic developments	Violence against civilians	Total (%)
Central Equatoria	101	5	7	3	34	115	265 (39.4)
Eastern Equatoria	9	2		2	2	28	43 (6.4)
Jonglei	19	2	1	3	2	44	71 (10.5)
Lakes	26		3	3	4	28	64 (9.5)
Northern Bahr el Ghazal	3			1		1	5 (0.7)
Unity	13				3	43	59 (8.8)
Upper Nile	16		2			9	27 (4.0)
Warrap	19				1	32	52 (7.7)
Western Bahr el Ghazal	27		1			20	48 (7.1)
Western Equatoria	13	1			2	23	39 (5.8)
Total (%)	246 (36.5)	10 (1.5)	14 (2.1)	12 (1.8)	48 (7.1)	343 (51.0)	673

Note. States based on ACLED definitions and data from September 2018–August 2019

#### Onchocerciasis mapping and MDA strategy

**Fig. 5:**
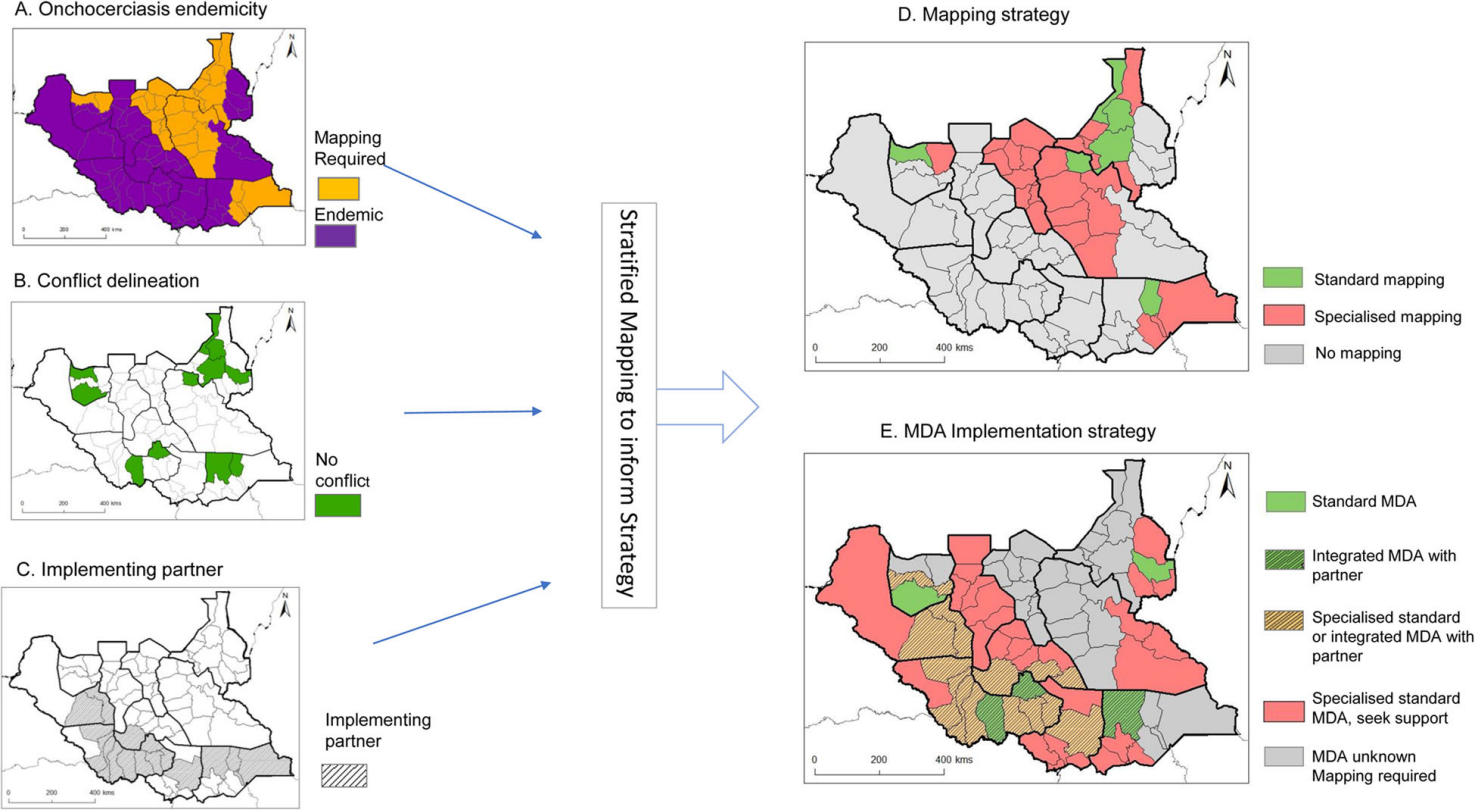
Onchocerciasis endemicity, conflict, implementing partners stratified to inform mapping and MDA implementation strategies in South Sudan. Data sources: ESPEN [23]*,* ACLED [24] and Administrative boundary source: HDX [30]

The combination of disease, conflict and implementing partner data was used to create maps to inform the mapping and MDA implementation strategies (Fig. 5a-d). Onchocerciasis elimination mapping was required in 31 districts across the states of Eastern Equatoria, Jonglei, Northern Bahr el Ghazal, Unity and Upper Nile (Fig. 5a). Of the 31 districts requiring mapping, most districts (*n* = 25) were classified as ‘not mapped/conflict’ indicating it was an unsafe zone and that specialised mapping adapted to the context and risk needed to be conducted. Onchocerciasis MDA implementation was required in all 48 districts mapped (Fig. 5a). The majority of districts were classified as ‘endemic/ conflict’ (*n* = 28) or ‘endemic/conflict/partner absent’ (*n* = 15) indicating an unsafe zone requiring specialised MDA, adapted to context and risk, with support from implementation partners in selected areas (Fig. 5a-d).

#### NNN conference – validation

The approach and case study were presented in two sessions of the annual NNN meeting in 2019; one a general workshop and the other the specific C&HE Cross-Cutting Group annual meeting [26]. Each session included 30–40 participants from 10+ NGOs, donors and representatives from ministries of health. The practicality and acceptability of the new methodology was assessed by placing participants into groups of 5–7 people, and paper copies of maps, conflict situation and NTD endemicity were provided. All participant groups were able to follow the method and present their results on the South Sudan case study. Following the smaller group work, feedback was sought through a larger group discussion. Overall, the participants found the methodological approach useful and felt it could be applied to different NTDs and programmatic activities within those NTDs beyond just surveys and MDA. It was recommended that the tool be used in additional countries.

## Discussion

The paper presents a new methodology for implementing safe and effective mapping and MDA strategies in NTD endemic countries with ongoing complex emergencies. It is the first time that NTD and conflict data have been brought together in this format to help inform national control and elimination programmes. This is important as conflict is a neglected topic, and events are rarely quantified in terms of the type, magnitude, location and impact, despite being referred to as a significant barrier to the progress of NTD programmes [11]. Conflict events can lead to political instability, divergence of funding, strains on health infrastructure and inadvertent mass migration, including refugees and IDPs, which can change the epidemiology of NTDs and perpetuate problems in their control, elimination and surveillance [6–9].

The development of this methodological approach stems from work by the NNN C&HE Cross-Cutting Group, who comprise a diverse range of NTD programme managers and researchers working in endemic countries experiencing complex emergencies [26]. The NNN C&HE Group used an international NTD NGO forum to present a case study on South Sudan to confirm the practicality and acceptability of the methodology, thereby advocating its use and roll-out to other countries and diseases. It has also successfully been piloted for LF and trachoma in other countries given the standard data available for all endemic countries on the ESPEN [23] and Global Trachoma [29] websites. Drawing on this extensive network of NTD expertise was critical as there is a lack of formal guidelines for NTD national programmes to use and plan activities within complex emergencies. This highlights the need for more formal discussions and protocol development with WHO on the challenges of complex emergencies, as done with other arising issues [31–33], and most recently with Covid-19, which has impacted the new NTD Roadmap 2021–2030 and initiated widespread international response and collaboration [11, 34, 35].

The availability of NTD programme and conflict data through online portals i.e. ESPEN, Global Trachoma Atlas and ACLED [23–25, 29] is ground-breaking and has enabled this first step to be developed. However, it is recognised that databases reporting this information may not have the full picture, and it is therefore critical to triangulate information. It is acknowledged that the additional use of this data may increase the workload on stretched programmes. Therefore, a nominated focal person may benefit from being trained to assess real-time, open-source digital platforms such as ACLED, and collate and map key data prior to programme activities. This will help to optimise time and human resources, as well as ensure safety for staff. A further consideration is that NTD data quality and completeness is variable and reporting systems are particularly fragile in conflict-prone countries [8]. Checking a country’s security status by engaging with government ministries and implementing partners operating within affected districts is essential. Local knowledge and trusted partners will often be the most useful and reliable source of information, which can be supplemented by other sources such as the Humanitarian Data Exchange [36] and Operational Portal Refugee Situations [37].

It is also important to consider what constitutes “secure” or a “risk” as this is subjective. Different groups may differ in their perception of what areas of the country are considered accessible for NTD programming. Some entities will be more risk adverse than others. Additionally, security is a fluid concept, with on-the-ground dynamics constantly changing. For this reason, programmes may find that districts that are considered “insecure” by one organisation, may be accessible by others given their local knowledge, experience and relationships within those districts, as evidenced by mapping conducted in areas previously or currently considered insecure [38, 39]. The humanitarian sector is increasingly using online geo-referenced data to support operations [40] and this needs to be embraced by the NTD community, with support for training of NTD personnel.

The SDGs include the ambition to end the epidemics of NTDs by 2030 [11, 22]. This cannot be achieved if NTD burdens are left unknown and unaddressed in countries recovering from or experiencing complex emergencies. Documenting and monitoring the impact is critical to improve our understanding and develop surveillance systems. It is understandable that security of staff must be taken into consideration when planning and conducting activities, including mapping; however, as our South Sudan case study shows, there are often districts within a country that can be accessible for baseline or impact mapping and implementation of programmatic interventions despite the country overall being labelled as “insecure”.

## Conclusion

The decision tree presented in this paper is an important first step in helping national NTD control and elimination programmes make informed decisions about mapping and MDA implementation in the context of complex emergencies. This practical methodological approach needs to be extended across all endemic conflict-affected countries in sub-Saharan Africa with support from national and international partners so that they can make progress towards reducing the burden of disease and meet the SDGs and NTD Roadmap targets of 2030.

## Supplementary Information

**Additional file 1 Table S1.** ACLED conflict types and their definitions

**Additional file 2 Table S2.** Country coverage rates 2018

**Additional file 3 Table S3.** South Sudan NTD endemicity, implementing partner and conflict status

## Data Availability

All data are available in the manuscript, additional files or the open access data sources referenced.

## Abbreviations

ACLED: Armed Conflict Location and Event Data
AFR: African region
ESPEN: Expanded Special Project for Elimination of Neglected Tropical Diseases
EU: Evaluation unit
IDPs: Internally displaced persons IU Implementation unit
LF: Lymphatic filariasis
NGO: Non-governmental organisation
NGDO: Non-governmental development organisation
NNN: Neglected Tropical Disease NGO Network
NTD: Neglected tropical diseases
MDA: Mass drug administration
PAC: Pre-school aged children
PC: Preventive chemotherapy
SAC: School-aged children
STH: Soil transmitted helminths
WHO: World Health Organization
