# Supplementary material for: Complex emergencies and the control and elimination of neglected tropical diseases in Africa: developing a practical approach for implementing safe and effective mapping and intervention strategies
Source: Confl Health. 2021 Mar 31;15:18. doi: 10.1186/s13031-021-00356-7 (PMC8010498; doi:10.1186/s13031-021-00356-7)
Supplement: Supplementary file 1 — Additional file 1 Table S1. ACLED conflict types and their definitions [file 13031_2021_356_MOESM1_ESM.pdf]

ACLED conflict types and their definitions

Conflict type	Definition
**Battles**	Involve state or non-state actors, engaging in armed conflict with possible attempts to claim or reclaim territory.
**Riots**	Involve violent demonstrations of rioters, either civilians or mobs, against state actors including military and police forces.
**Protests**	Include both peaceful and violent protesting, whereby protests may progress to an intervention by non-state actors or result in excessive force used by state actors against the protesting.
**Strategic developments**	Broadly includes the following sub-event types – agreement between opposing parties; arrests; change to group/activity; disrupted weapons use; looting/property destruction; non-violent transfer of territory.
**Explosions / remote violence**	Encompasses various acts of violence involving the use of explosive materials, including: air/drone strikes; chemical weapons; grenades; missile attacks; remotely-detonated explosives' landmines; suicide bombings.
**Violence against civilians**	Self-explanatory, but includes attacks on un-armed civilians, abductions/forced disappearances, and sexual violence.

Data available https://www.acleddata.com
